# Macroevolution in axial morphospace: innovations accompanying the transition to marine environments in elapid snakes

**DOI:** 10.1098/rsos.221087

**Published:** 2022-12-21

**Authors:** Emma Sherratt, Tamika Nash-Hahn, James H. Nankivell, Arne R. Rasmussen, Paul M. Hampton, Kate L. Sanders

**Affiliations:** ^1^ School of Biological Sciences, The University of Adelaide, Adelaide, South Australia 5005, Australia; ^2^ South Australian Museum, North Terrace, Adelaide, South Australia 5000, Australia; ^3^ The Royal Danish Academy, Institute of Conservation, 1435 Copenhagen, Denmark; ^4^ Department of Biology, Colorado Mesa University, Grand Junction, CO 81501, USA

**Keywords:** evolution, innovation, development, axial column, regionalization, sea snake

## Abstract

Sea snakes in the *Hydrophis-Microcephalophis* clade (Elapidae) show exceptional body shape variation along a continuum from similar forebody and hindbody girths, to dramatically reduced girths of the forebody relative to hindbody. The latter is associated with specializations on burrowing prey. This variation underpins high sympatric diversity and species richness and is not shared by other marine (or terrestrial) snakes. Here, we examined a hypothesis that macroevolutionary changes in axial development contribute to the propensity, at clade level, for body shape change. We quantified variation in the number and size of vertebrae in two body regions (pre- and post-apex of the heart) for approximately 94 terrestrial and marine elapids. We found *Hydrophis-Microcephalophis* exhibit increased rates of vertebral evolution in the pre- versus post-apex regions compared to all other Australasian elapids. Unlike other marine and terrestrial elapids, axial elongation in *Hydrophis-Microcephalophis* occurs via the preferential addition of vertebrae pre-heart apex, which is the region that undergoes concomitant shifts in vertebral number and size during transitions along the relative fore- to hindbody girth axis. We suggest that this macroevolutionary developmental change has potentially acted as a key innovation in *Hydrophis-Microcephalophis* by facilitating novel (especially burrowing) prey specializations that are not shared with other marine snakes.

## 1. Introduction

Radiations that display great species richness and morphological diversity are found throughout the tree of life and have provided vivid insights into the mechanisms governing biodiversity [1,2]. A fundamental expectation is that heterogeneous patterns of clade diversity are linked to clade age—older groups have simply had more time to accumulate diversity than younger groups (e.g. [3]). However, diversity patterns can also be shaped by disparities in the ecological carrying capacities of clades and/or unequal diversification (speciation minus extinction) rates [4,5]. In these instances, elevated species richness is often associated with geographical or ecological opportunities, such as transitions to new environments or the acquisition of key innovations [6] that facilitate expansions into new niches.

Body shape and size are tightly linked to ecological traits and life history in the 390 species of terrestrial, amphibious and marine elapid snakes [7]. Within this radiation, the *Hydrophis* and *Microcephalophis* clade of approximately 50 fully marine sea snakes (Elapidae: Hydrophiinae) shows exceptionally high body shape disparity and sympatric species richness. Assemblages contain diverse tropic specialists differentiated along a continuum from similar forebody (pre-apex of the heart) relative to hindbody (post-apex) girths, to dramatically reduced forebody relative to hindbody girths [8–10]. Predators of crevice eels or spiny fishes typically have similar fore- versus hindbody girths, whereas ecomorphs with reduced fore- versus hindbody girths (‘microcephalic’ forms) specialize on burrowing fish prey [10]. Both *Microcephalophis* species and almost a third of *Hydrophis* have reduced forebody girths and feed almost exclusively on burrowing prey. However, while this ecomorph has evolved many times in *Microcephalophis-Hydrophis*, it is not found in any other marine (or terrestrial) snakes, including the fully marine and contemporaneous *Aipysurus-Emydocephalus* clade.

In *Hydrophis-Microcephalophis*, variation in relative body girth is underpinned by extreme heterogeneity in vertebral number and size along the axial skeleton. We have shown previously that the microcephalic specialists of burrowing prey have proportionally more vertebrae in the forebody (pre-apex of the heart region) compared to other species of this marine clade [11]. This is facilitated by the forebody vertebrae being relatively smaller than those of the hindbody (post-apex region) [12]. Therefore, these species break the correlation between vertebral number and body length seen in many other snakes and elongate vertebrates (pleomerism *sensu* [13]). Further postnatal ontogenetic changes in these species cause their hindbodies to reach greater girths than their forebodies [11]. These observations imply that coordinated changes during vertebral development (somitogenesis) and homeotic (*Hox* gene) regulation underlie the evolutionary development of body shape changes in *Hydrophis* and *Microcephalophis*. Several previous studies have shown how heterochronic changes in somitogenesis have shaped axial diversity in snakes and other vertebrates [14–17]. Much less attention has been given to homeotic changes along the snake axial column, which is nearly homogeneous in comparison to most vertebrates and lacks distinct anatomical landmarks. However, three distinct (cervical, lumbar and thoracic) regions of the pre-cloacal vertebrae have been identified [18,19], providing important evidence of *Hox* transitions along the pre-cloacal axis in snakes [19]. More recently, the heart was proposed to align near the boundary of the fore- and hindbody thoracic regions [11,12,20], and this has been substantiated by reports of a transitional boundary in vertebral shape in the colubrid snake *Thamnophis* [21].

In this paper, we examine macroevolutionary patterns of vertebral development in fore- versus hindbody regions of elapid snakes. This is to examine a hypothesis that clade-wide differences in axial development have contributed to the greater propensity for body shape diversification in *Hydrophis-Microcephalophis* compared to other marine and terrestrial elapids. For 94 elapid species, we quantified patterns of variation in vertebral counts (pre- and post-apex of the heart) and characterized shapes of intracolumnar profiles. This is the first study to link axial diversification and ecological niche shifts to clade-wide adaptive potential in snakes.

## 2. Material and methods

### 2.1. Vertebral number: samples and measurements

We sampled 275 alcohol-preserved adult specimens representing 94 species of terrestrial (*n* = 51), fully marine (*n* = 40) and semi-aquatic (*n* = 3) ecologies from the Elapidae family (electronic supplementary material, table S1). Species were chosen from the Australasian region to capture the diversity in number of pre-cloacal vertebrae. Details of specimens studied and the museums from which they were sourced are given in the electronic supplementary material, table S1. Sex was not considered in this study, but tail vertebrae were omitted because they show the most sexual dimorphism. Data for marine species were taken from [11]. Counts of vertebrae were made from ventral scales for the terrestrial species, and *Aipysurus* + *Emydocephalus* species group, since they display a 1 : 1 ratio for pre-cloacal vertebrae [22]. Heart position was found through small ventral incisions and noted based upon vertebral number counted from behind the head to posterior-most point of heart. Other marine species were examined using X-rays since they do not display the ventral scale correspondence (details of X-rays are given below). Metal pins were placed into the preserved specimens at the posterior-most point of the heart and the cloaca to be visible in X-rays (example shown in the electronic supplementary material, figure S1). Counts started at the first vertebra attaching to the skull and proceeded to the last vertebra anterior to the cloaca.

Digital X-rays were made using two systems: small specimens were imaged using the Faxitron LX-60 machine at University of Adelaide Health and Medical Sciences facility, and for large specimens, we used a Siemens Multix Fusion Max machine at Dr. Jones and Partners Medical Imaging, Adelaide (electronic supplementary material, figure S1). Both systems save the X-rays as a DICOM format with size embedded into the file, such that measurements are scaled automatically.

Absolute body size and number of pre-cloacal vertebrae differ greatly among the elapid species we sampled; for example, among the smallest species is a terrestrial/fossorial species, *Simoselaps bertholdi*, at 23.5 cm neck to cloaca with 124 pre-cloacal vertebrae, and among the largest is a terrestrial species, *Oxyuranus scutellatus*, at 146.5 cm and 237 vertebrae. But length and number of vertebrae are not always correlated in elapid snakes [22,23]; for example, *Toxicocalamus preussi* is one of the smaller species at 55 cm with the most pre-cloaca vertebrae in the dataset (325). Since snakes have indeterminate growth, and museum samples may be skewed to smaller individuals, absolute body size was not considered further; instead relative vertebra size was used (actual vertebra size divided by body length, see below), which is known to remain consistent during ontogeny in some elapids [12] (but see [24,25]).

### 2.2. Intracolumnar vertebral size: samples and measurements

To capture variation in intracolumnar vertebral size, we sampled one adult of each species for a subset of 61 species of terrestrial (*n* = 27), fully marine (*n* = 31) and semi-aquatic (*n* = 3) elapid snakes (electronic supplementary material, table S2). Sampling one individual per species is sufficient in this instance because there is no appreciable difference in vertebral column intracolumnar profiles among adults within species (electronic supplementary material, figure S2), nor during postnatal growth [12]. Vertebrae size was examined by measuring the length of every vertebra from the first pre-cloacal vertebra after the atlas to the vertebra anterior to the cloaca [11]. Tail vertebrae were not examined in this study because they are known to exhibit sexual dimorphism (e.g. [26]). We measured the length of each vertebra from the X-rays using the ‘multipoint tool’ in ImageJ v.1.52i [27]: landmarks were placed medially along the vertebral column at the anterior limit of the centrum of each vertebra. Coordinates (*x*,*y*) of the landmarks in millimetres were exported into the R statistical environment v.4.0.5 [28] and inter-landmark distances were calculated by applying the Pythagorean theorem between sequential coordinate points. Vertebral width could not be measured due to the changing orientation of the vertebral column (axial torsion) resulting from specimen preservation.

### 2.3. Phylogenetic hypothesis

We built a consensus phylogenetic tree of elapids to perform comparative analysis. Molecular data were obtained for 183 species of elapid using the mitochondrial 12S, 16S, ND4 and cytochrome b genes and the nuclear C-mos, RAG-1 and RAG-2 genes. The bulk of the alignment was obtained from [29]; however, we removed *Aipysurus pooleorum* as the genbank sequences were a composite of *A. pooleorum* and *A. foliosquama*. We added *Acanthophis laevis*, *Acanthophis pyrrhus*,* Hoplocephalus bungaroides*, *Neelaps bimaculatus*, *Pseudonaja inframacula* and *Pseudonaja nuchalis* from genbank sequences. The sea snake taxa *Aipysurus tenuis* and *Emydocephalus orarius* were added from electronic supplementary material in [30], while new ND4 sequence was obtained for *Demansia reticulata* (Genbank number OP957439) using the protocols in [31]. Additional taxa were aligned and checked by eye in Geneious Prime v. 2022.0.1 (https://www.geneious.com) resulting in a final alignment of 9078 bp with 183 terminal taxa.

We initially used the partitioning scheme of [29]; however, due to issues with reaching convergence we used simpler models of sequence evolution. The final partitioning scheme consisted of the following partitions: (i) nuclear coding regions, codons 1 + 2 – HKYig; (ii) nuclear coding regions, codon 3 – HKYig; (iii) mitochondrial coding regions, codon 1 – HKYig; (iv) mitochondrial coding regions, codon 2 – HKYig; (v) mitochondrial regions, codon 3 – HKYg; (vi) 12S rRNA – HKYig and (vii) 16S rRNA – HKYig. Phylogeny and dates were reconstructed using BEAST v. 2.6.6 with dates calibrated using the same nodes as [29]. Clock and tree models were linked with a strict clock and a Yule tree model was selected. The Markov chain Monte Carlo was run for 10 000 000 generations with trees sampled every 1000 states. Convergence was checked using Tracer v.1.7.2 [32] and a burn in of 25% and effective sample size values of greater than 100 were reached for most parameters before burn in. Maximum clade credibility trees were produced from the remaining 7500 trees using TreeAnnotator v.2.6.6 [33]. The tree was pruned to 90 taxa included in this study using the ‘drop.tip’ function in *ape* R package v.5.6-2 [34], where *Denisonia maculata* was substituted for *Denisonia devisi*. Taxa without a suitable substitution were omitted from comparative analyses: *Brachyurophis fasciolatus*,* Hoplocephalus stephensi*,* Salmonelaps par* and *Hydrophis melanosoma*.

We divided the tree into four groups based upon ecological niche and phylogenetic relatedness [31]: terrestrial species; the monophyletic group of marine species (*Aipysurus* and *Emydocephalus*); the semi-aquatic species (*Hydrelaps darwiniensis*,* Ephalophis greyae* and* Parahydrophis mertoni*); and the other monophyletic group of marine species (*Hydrophis* and *Microcephalophis*). Note that the semi-aquatic species render the marine clade paraphyletic.

### 2.4. Statistical analyses

To assess how regionalization may affect the vertebral profile composition, we subdivided the counts of pre-cloacal vertebrae into number in the forebody (anterior to the apex of the heart) and the hindbody (posterior to the apex of the heart), where heart position is taken as the vertebra position aligned to the posterior apex of the heart. We also calculated the proportion of the vertebrae in the forebody, as a ratio of pre-apex vertebrae to the number of pre-cloacal vertebrae. Macroevolutionary patterns of intracolumnar variation were examined by mapping the proportion of forebody vertebrae onto the consensus phylogenetic tree of elapids. Ancestral state estimation was done using maximum likelihood, implemented with the ‘fastAnc’ function in *phytools* R package. These were used to plot a traitgram, projecting the phylogenetic tree into a space defined by the proportion of vertebrae above the heart and relative time since the root of the tree.

Rates of morphological evolution between numbers of vertebrae in the fore- and hindbody among the four species groups were estimated using a Brownian motion model for high-dimensional data [35] implemented with the ‘compare.evol.rates’ function in *geomorph* R package v.4.0.4 [36] and evaluated for statistical significance through permutation (1000 iterations). Phylogenetic signal of the number of pre-cloacal vertebrae, the number of pre- and post-apex vertebrae, and the proportion of vertebrae in the forebody were evaluated using the K-statistic [37] implemented with ‘physignal’ in *geomorph* R package.

To study intracolumnar variation, we plotted sequentially vertebral size against position, to create intracolumnar ‘profiles’ (e.g. [11,18,19,25,38–41]). In order to compare intracolumnar profile shapes among species we performed two standardizing procedures. First, we accounted for absolute size variation among species by calculating relative vertebral size: vertebra length divided by the sum of all vertebra lengths (approximately equivalent to total body length from neck to cloaca). Owing to differential growth of vertebrae along the axial column, the resulting intracolumnar profile is curved with a positive inflection point following an approximate polynomial of the fourth degree (4°). So, we accounted for differences in the number of vertebrae among species by fitting 4° polynomials to each profile using the *stats* R package functions ‘lm’ and ‘poly’ (arguments: degree = 4, raw = TRUE), generating 100 equally spaced predicted points along each profile. The profiles are plotted as a curved line with positive inflection on a two-dimensional plot, where the *y*-axis is relative vertebra size and the *x*-axis is vertebra number (index) (e.g. [12]) (electronic supplementary material, figure S3). The curves of all species are plotted in the electronic supplementary material, figure S4.

The predicted values defining the profiles were appended to the new vertebra lengths, then ordinated with a principal component analysis (PCA) (‘gm.prcomp’ R function in *geomorph*, scale = TRUE) to visualize similarity in profiles among species. The first two PC axes were visualized with a scatterplot, with points representing each species and point size being relative to the number of pre-cloacal vertebrae. Profile curve shapes representing the minima and maxima of the PC axes were plotted to illustrate the shape variation described by each axis. Then to examine how regionalization may influence profile shape among species, we divided the profiles into two parts, forebody (pre-apex of heart) and hindbody (post-apex) and applied the same polynomial approach to fit 15 and 20 predicted points to the regions, respectively. These data were appended to recreate a full profile, ordinated with PCA and visualized as above.

A phylogenetic generalized least-squares (PGLS) analysis was used to test whether heart position predicts profile shape, implemented with the ‘procD.pgls’ function in *geomorph*. Phylogenetic relationships were inferred from the consensus phylogenetic tree of elapids, pruned to 56 species of the 61 sampled. We examined whether rates of morphological evolution in profile shape differed among the four species groups using the ‘compare.evol.rates’ function in *geomorph* R package and evaluated for statistical significance through permutation (1000 iterations). Phylogenetic signals of the shape of the profiles, with and without defined heart position, were evaluated using a multivariate extension of the K-statistic, *K*_mult_ [42].

## 3. Results

**Figure 1.**
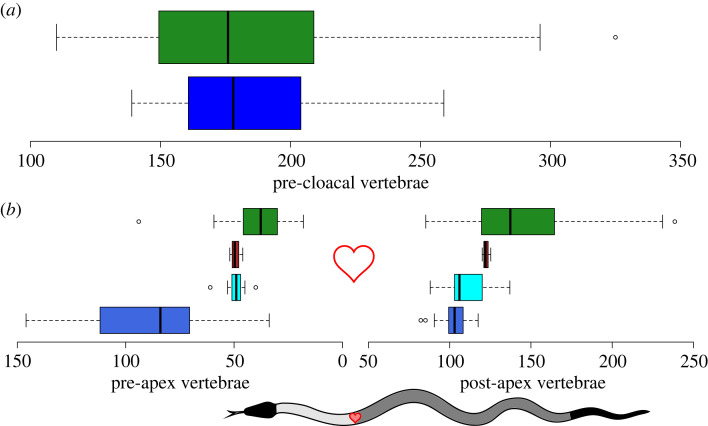
Summary of pre-cloacal vertebrae between terrestrial and marine species. (*a*) Total number of pre-cloacal vertebrae in terrestrial (green, *n* = 51) and marine (blue, *n* = 43) species of elapids. (*b*) Number of vertebrae in the forebody (pre-apex of the heart, left) and the number in the hindbody (post-apex excluding tail, right) across the terrestrial species (green) and the three main groups of marine species: brown is semi-aquatic species (*Ephalophis*, *Hydrelaps* and *Parahydrophis*), cyan is *Aipysurus* + *Emydocephalus* clade and royal blue is *Hydrophis* + *Microcephalophis* clade. Schematic snake shows fore- and hindbody regions and heart for reference.

Marine elapids have less diversity in the number of pre-cloacal vertebrae compared to terrestrial elapids, but the two groups' averages are not appreciably different (figure 1*a*): marine species have 182.8 (±28.42) pre-cloacal vertebrae and terrestrial have 181.4 (±43.48). However, the two groups differ substantially in where these vertebrae are positioned along the body (figure 1*b*): terrestrial species and the *Aipysurus* + *Emydocephalus* clade vary more in the number of post-apex vertebrae (hindbody), while the *Hydrophis* + *Microcephalophis* clade vary in the number of pre-apex vertebrae (forebody). The three semi-aquatic species have consistently similar numbers of vertebrae in both body regions.

**Figure 2.**
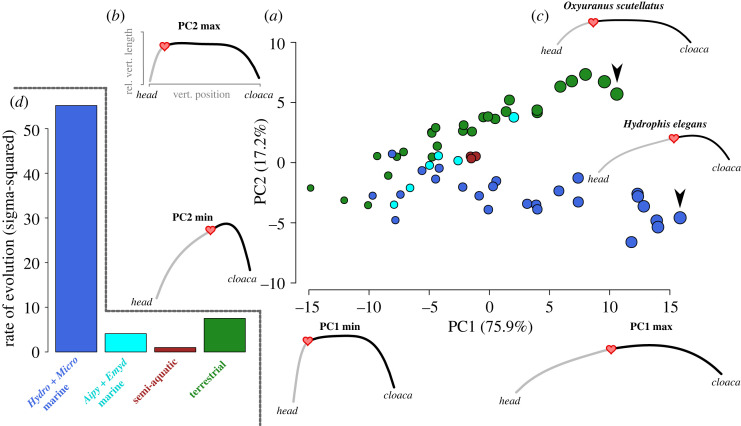
(*a*) Principal component (PC) 1 and 2 scatterplot (75.9% + 17.2% = 93.1%) of the standardized intracolumnar profile shapes for pre- and post-apex regions, showing the diversity of intracolumnar profile shape including heart apex position for 61 species of elapids. Each point is scaled to number of vertebrae in the profile and coloured as in figure 1 (green is terrestrial species; brown is semi-aquatic species *Ephalophis*, *Hydrelaps* and *Parahydrophis*; cyan is *Aipysurus* and *Emydocephalus*; royal blue is *Hydrophis* and *Microcephalophis*). (*b*) Intracolumnar profile shapes representing minimum and maximum PC scores for each axis are given below and beside the axes (labelled PC1 min, PC1 max etc.), and are depicted as a 4° polynomial curve with positive inflection. This is made by plotting relative vertebrae size on *y*-axis and vertebrae position on *x*-axis (see §2 for details), from head (left) to cloaca (right). Heart position is marked by a red heart, and the profile shapes are coloured so that grey is the forebody (pre-apex of the heart) and black is hindbody (post-apex) region. (*c*) The profile shape of two species at the extremes of the terrestrial and marine groups is also depicted. (*d*) Bar plot of the rates of evolution (***σ***^2^) for intracolumnar profile shape among ecological groups and coloured as in (*a*).

There is also great shape variability in the intracolumnar profiles of the 61 species of elapid snakes, as demonstrated by their distribution within an axial morphospace defined by two axes (total 93% of shape variance; figure 2). Within-species variation is minimal by comparison (electronic supplementary material, figure S2). Among-species variation in the number of pre-cloacal vertebrae (demonstrated by the size of the plotted point in the PCA scatterplot) varies along PC1. The profile shape change along PC1 is from a steeply curved, short profile (negative PC scores) to a long, wide profile (positive PC scores). PC2 describes asymmetry in the shape of the profile curve and is driven by the heart position: positive PC2 scores describe species with the largest vertebrae positioned anteriorly and having a short forebody region, while those where the largest vertebrae are more posterior have long hindbody regions (negative PC2 scores). Within this axial morphospace, terrestrial taxa follow a narrow trajectory along an axis of increasing number of vertebrae. Marine species of the *Hydrophis* + *Microcephalophis* clade diverge on a separate trajectory, and semi-aquatic taxa occupy an intermediate position. Members of the *Aipysurus* + *Emydocephalus* clade species appear to follow the trajectory of terrestrial species, except one species (*A. duboisii*), which follows the other marine. *Hydrophis platura*, the only pelagic sea snake, is also an exception and falls inside the terrestrial trajectory. Three other marine *Hydrophis* species (*H. curtus*,* H. viperinus* and* H. annandalei*) also tend to follow the trajectory of the majority of *Aipysurus* species.

Intracolumnar profile shape is also strongly correlated with the position of the heart (PGLS *R*^2^ = 0.41, *F*_1,54_ = 39.06, *p* = 0.001; figure 2). When heart position is not included in the profile shape, the axial morphospace is dominated by the first axis (91.5% + 5.7% = 97.2%), along which both terrestrial and marine species are distributed (electronic supplementary material, figure S4) and relates to profile length. On PC2 terrestrial and marine species occupy parallel trajectories, differentiated by asymmetry in the profile shape.

**Figure 3.**
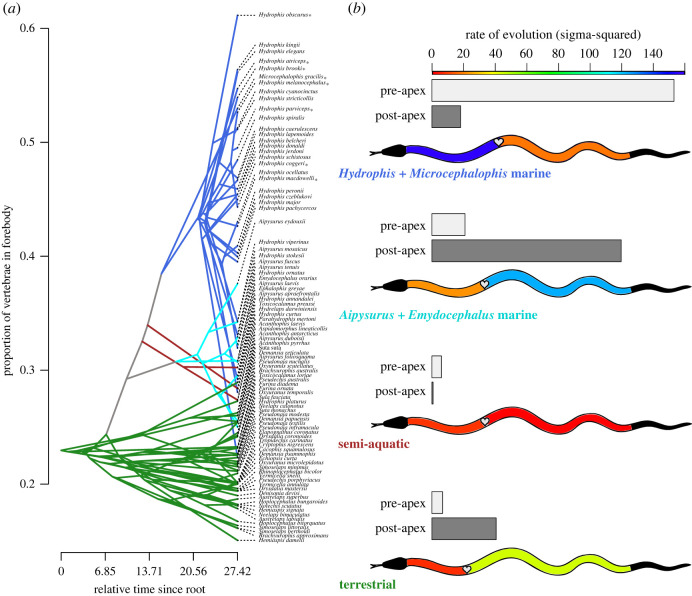
(*a*) Traitgram, showing the proportion of vertebrae in the forebody mapped onto the consensus tree of 90 species. The four groups are coloured as in figure 1. Taxa marked with asterisk (*) are eel-specialist microcephalic species, which all have very high proportions of vertebrae in the forebody. See electronic supplementary material, figure S5, for an alternative method for visualizing these data on the tree. (*b*) Rates of evolution (***σ***^2^) for pre-apex of the heart (forebody) vertebral counts and post-apex (hindbody) vertebral counts among groups. Schematic snake shows rate in each region coloured along a heatmap gradient.

Mapping the proportion of vertebrae in the forebody onto a phylogeny reveals a distinct evolutionary pattern (figure 3*a*): marine species evolved to have a higher proportion of vertebrae in the forebody early in their radiation, and the ancestor of all *Hydrophis* with *Microcephalophis* is estimated to have a substantially higher proportion than that of the *Aipysurus* + *Emydocephalus* clade. The species that are eel-specialists with the distinctive ‘microcephalic’ body shape (asterisk in figure 3*a*) show convergent evolution towards the highest proportion of vertebrae in the forebody. However, six species of *Hydrophis* have apparently evolved away from the clade average to have similarly lower proportions seen in *Aipysurus*: *H. platura*,* H. annandalei*,* H. curtus*,* H. ornatus*,* H. stokesii* and *H. viperinus*. One terrestrial species (*Toxicocalamus preussi*) is an outlier with a proportion similar to the semi-aquatic species, but the reason for this is unknown as this species has fossorial habits.

Rates of morphological evolution differ markedly between groups and pre- and post-apex regions (table 1; figure 3*b*). Rates are higher in the hindbody region compared to the forebody for all groups except the *Hydrophis* + *Microcephalophis* clade, which shows the opposite pattern. The rate of evolution of forebody vertebrae in the *Hydrophis* + *Microcephalophis* clade is 7 times higher than among species of the *Aipysurus* clade and 22 times higher than among terrestrial species. Terrestrial species have a rate of evolution in hindbody vertebrae that is 2.2 times higher, and species of the *Aipysurus* clade have a rate of evolution in hindbody vertebrae that is 6 times higher, than among species of the *Hydrophis* + *Microcephalophis* group. This indicates that the forebody region is evolving faster in the *Hydrophis* + *Microcephalophis* clade, and the hindbody region is evolving faster in terrestrial species and species of the *Aipysurus* clade.

**Table 1.** Rates of morphological evolution (***σ***^2^) compared among the four species groups for each vertebral column trait. Observed-rate ratio is the ratio of maximum to minimum net evolutionary rates; in all traits, the maximum rate was found in the *Hydrophis* + *Microcephalophis* group and the minimum in the semi-aquatic group. Figure 3*b* visualizes the observed rates for each group for pre- and post-apex regions. Effect sizes and *p*-values based on 1000 random permutations.

trait	observed rate ratio	effect size	*p*-value
profile shape	51.915	4.8301	0.001
profile shape with apex position	56.3829	4.1901	0.001
pre-cloacal vertebrae	52.7545	2.2859	0.007
pre-apex vertebrae	25.2725	2.0804	0.021
post-apex vertebrae	155.596	2.4414	0.003
proportion of vertebrae in forebody	28.0636	2.117	0.019

Phylogenetic signal is highly variable across the measured vertebral traits (table 2). There is a significant phylogenetic signal in profile shape with heart apex position, but much lower than expected under a model of Brownian motion (*K* < 1). Without heart position, profile shape does not have a significant phylogenetic signal. With respect to the number of vertebrae, all tests are significant but with very different estimates of *K*; post-apex vertebrae have more phylogenetic signal than expected under a model of Brownian motion (*K* > 1).

**Table 2.** Phylogenetic signal (Blomberg's *K* and *K*_mult_) for the vertebral column traits. Effect sizes and *p*-values based on 1000 random permutations.

trait	*K*/*K*_mult_	effect size	*p*-value
profile shape	0.391	1.1577	0.117
profile shape with apex position	0.468	1.9596	0.03
pre-cloacal vertebrae	0.553	5.1258	0.001
pre-apex vertebrae	0.701	5.0419	0.001
post-apex vertebrae	1.058	5.5313	0.001
proportion of vertebrae in forebody	1.416	10.7376	0.001

## 4. Discussion

Our study of morphological variation in the axial columns of elapid snakes has revealed striking macroevolutionary developmental patterns. In the terrestrial species and fully marine *Aipysurus*-*Emydocephalus* clade, vertebral numbers in the hindbody vary significantly more and evolve at a faster rate than vertebral numbers in the forebody region. This pattern is reversed in *Hydrophis* and its sister lineage *Microcephalophis*. In both microcephalic and non-microcephalic members of this clade, vertebral counts and lengths show significantly higher variation and faster evolutionary rates in the forebody than in the hindbody region. These results have important implications for understanding how innovations and constraints in axial column development may have shaped snake diversification.

### 4.1. Anterior axial development as a key innovation in sea snakes

High interspecific variation in body length and pre-cloacal vertebral counts is found across the whole elapid radiation [43]. Yet substantial change along the relative girth axis is characteristic of only *Hydrophis* and *Microcephalophis* and is tightly linked to trophic divergence in these species [8,10]. There are obvious locomotory constraints that must limit shape variation along the pre-cloacal axis of terrestrial and amphibious snakes. However, it is conspicuous that while the fully marine *Aipysurus-Emydocephalus* clade shows substantial variation in body length and girth, and head size and shape, none of these species has reduced fore- to hindbody girths or substantial proportions of burrowing prey in their diets [8,10]. It is possible that *Aipysurus-Emydocephalus* species have responded to different ecological selection pressures on body shape. However, we consider this to be unlikely given that the *Aipysurus-Emydocephalus* and *Hydrophis-Microcephalophis* clades have diversified contemporaneously across overlapping geographical regions, occupy many of same habitats and share other (crevice-sheltering and open-water) prey resources [30,44].

Shifting patterns of axial morphological development provide an alternative, and plausible, explanation for the disparity in body shape diversity of these marine clades. *Aipysurus-Emydocephalus* and the ancestral terrestrial species share a similar pattern of axial development; vertebral count is increased primarily in the hindbody region, and newly added vertebrae have similar lengths relative to the forebody vertebrae. This pattern is sharply reversed in most *Hydrophis-Microcephalophis*. In these species, axial extension occurs primarily in the forebody region and is accompanied by a reduction in vertebral length in lineages that undergo transitions to reduced relative forebody girth. The consequences of these patterns for constraining or facilitating body shape change in the two clades are best understood in the context of the clock and wavefront model of somitogenesis [15,16]. Here, a molecular oscillator controls whether larger numbers of smaller vertebrae, or fewer larger vertebrae, are produced for an axis of the same length [14,17]. Under this model, heterochronic changes to somitogenesis in the forebody region may provide *Hydrophis-Microcephalophis* with an efficient pathway to developing burrowing-prey specializations that is not available to *Aipysurus-Emydocephalus*. This is because a concomitant increase in vertebral number and reduction in vertebral size is needed to develop highly flexible, whip-like forebodies for extracting large aggressive eels from their burrows. In *Aipysurus-Emydocephalus*, delayed growth of the fore- versus hindbody might reduce their relative forebody girth, but without a concomitant increase in the number of vertebrae in this region, this would result in a slender-girthed but short and inflexible forebody, less adept to extract prey from narrow burrows.

It is clear that repeated origins of microcephaly have strongly contributed to the elevated rate of pre- versus post-apex change in *Hydrophis-Microcephalophis* (figure 3*a*). However, in this clade, as many non-microcephalic (as microcephalic) lineages show an increased proportion of vertebrae in the forebody, and this includes species that are unlikely to represent reversals from microcephalic ancestors (e.g. *H. kingii* and *H. ocellatus*; figure 3*a*). This suggests that the patterns found here are not merely the result of multiple separate morphological transitions, but instead reflect a macroevolutionary (clade-wide) shift in axial development. This shift either might have been associated with the initial transition to microcephaly, or alternatively may have pre-dated the first origin of microcephaly and facilitated subsequent transitions to this phenotype. It also remains to be determined how the loss of the one-to-one relationship between ventral (belly) scales and vertebrae in *Hydrophis*-*Microcephalophis (*e.g. [45]) might be linked to axial development, if at all. *Aipysurus-Emydocephalus* have retained the ancestral coupling of ventral scales and vertebrae, but nonetheless show very rapid changes in numbers of (predominantly hindbody) vertebrae.

Key innovations are traits that allow a taxon to access new ecological resources and, as a consequence, their acquisition can increase species richness relative to sister lineages [46]. Rigorous tests of the potential role of particular traits in species diversification require more replication than is available in our single clade of sea snakes (see [47]). Nonetheless, patterns of species diversity in *Hydrophis*-*Microcephalophis* suggest this clade may have a higher ecological carrying capacity, consistent with the key innovation hypothesis. Firstly, *Hydrophis*-*Microcephalophis* contains 49 species, while 12 species are recognized in *Aipysurus-Emydocephalus*, which likely also harbours fewer undescribed species. Where the two groups overlap in Australia and Southeast Asia, their maximum sympatric species richness varies by 12 *Hydrophis*-*Microcephalophis* to six *Aipysurus-Emydocephalus.* Co-occurring *Hydrophis*-*Microcephalophis* are characterized by disparate fore- versus hindbody girths and correspondingly partitioned diets. Similar fore- versus hindbody girths are typical of specialist predators of crevice eels (e.g. long-bodied species such as *H. cyanocinctus* in Asia and *H. elegans* in Australia) or spiny fishes (e.g. shorter bodied *H. viperinus* in Asia and *H. major* in Australia). Reduced fore- versus hindbody girths are associated with specializations on burrowing prey; snakes with moderately reduced forebody girths often specialize on burrowing gobies and goby-like fishes (e.g. *H. peronii*), and co-occur with species that have extremely reduced forebody girths and exclusively hunt eels in burrows (e.g. *H. atriceps*).

Each of these major ecomorphs has evolved multiple times in all major ocean basins [48]. However, species with reduced forebody girths and burrowing prey account for 28% of the total species richness of *Hydrophis*-*Microcephalophis* and have evolved independently in all major lineages within this clade. Although the evolution of key innovations is not always accompanied by accelerated speciation rates, this might be expected given the young age of the clades studied here. The *Hydrophis* crown group exhibits three-fold higher lineage diversification rates compared to other elapids. However, a phylogenetic lag of approximately seven million years separates *Hydrophis* crown group from its sister lineage *Microcephalophis.* This lag is not easily explained by biogeographic factors or events given that both lineages are widely distributed in the Indo-West Pacific.

### 4.2. Relationship between body elongation and cardiac physiology in snakes

Relative heart position is evolutionarily labile in snakes, shifting in position along the elongate body cavity, presumably because there is no diaphragm to constrain it. The heart, due to its role in circulating blood, is also more strongly influenced by gravity than other internal organs [49]. Previous studies have used measured distances to show that aquatic species have more centrally placed hearts compared to terrestrial species [50–53]. When heart position is measured as a heart-to-head distance, *Hydrophis* species have their heart at 28–42% of the total body length, compared to 23–26% in *Aipysurus-Emydocephalus* and the semi-aquatic *Hydrelaps darwiniensis*, and 15–23% in the terrestrial elapids [51]. The amphibious marine *Laticauda* (not sampled here) have heart positions at 21–36% of their body length [51]. The more central heart position of aquatic snakes has been linked to a release from gravitational constraints [51] and energetic efficiency [52]. Centrally placed hearts have also been observed in ground-dwelling viperids, which are expected to be less sensitive (than arboreal species) to the effects of gravity on circulation [49].

The results of the present paper are consistent with these previous studies. However, heart position can also be considered with respect to its position along the axial skeleton [10–12,20,54]. There is strong evidence from vertebral shape variation that the heart aligns with a conspicuous transitional boundary along the axial column in two species of colubrid snake (*Thamnophis*) [21]. In both species, Hampton *et al*. [21] found a statistically distinct morphological transition at approximately 17% of the pre-cloacal vertebral column, close to the position of the apex of the heart. By using this approach, it is possible to shift the dialogue from pattern and cause, to mechanism. In answer to the question of *how* do aquatic snakes have a more centrally placed heart, we previously demonstrated the developmental mechanism by which the heart becomes more centrally positioned in Hydrophiinae snakes [11,12]. Here we provide the evolutionary mechanism; a preferential addition of vertebrae in the forebody (pre-apex) region during body elongation underlies the apparent diversity in heart positions among aquatic elapids.

Further research is needed into the number of pre-cloacal vertebrae, pre- and post-apex of the heart, in *Acrochordus* (Acrochordidae), *Laticauda* (Elapidae), aquatic taxa of Homalopsidae and lesser known aquatic species (e.g. [55]) to understand whether the pattern of vertebral number between regions found in Hydrophiinae has been replicated in these unrelated aquatic lineages. Furthermore, an avenue of important research lies in assessing whether a centrally placed heart is an energetically efficient adaptation in other elongate aquatic animals, such as eels. Axial elongation through size and number of vertebrae has also been identified as an evolutionary mechanism behind body shape diversity of eels and other elongate fishes [56,57]. Heart position in eels has received much less scientific attention, however, but it is noted that *Moringua* eels have a more caudally shifted heart relative to other eels [58], and worth further investigation. Finally, further investigations are encouraged into how other organs are displaced along the body relative to vertebral positioning during the transition to aquatic lifestyles (e.g. [20,59]) and during ontogeny (e.g. [60]) as a means to better understand the developmental mechanism underlying regionalization of the snake's Bauplan.

## Data Availability

Morphological data and R code are available from the Dryad Digital Repository: https://doi.org/10.5061/dryad.6m905qg31 [61]. X-rays are available on Morphosource.org (project ID: 000445258). The data are provided in the electronic supplementary material [62].
